# Economic evaluation of a leprosy innovation project in Northern Nigeria: cost-effectiveness analysis

**DOI:** 10.1186/s12962-022-00393-w

**Published:** 2022-10-29

**Authors:** Charles C. Ezenduka, Abudulahi Namadi, Dahiru Tahir, Uzoma Nwosu, Shuaibu N. Musa

**Affiliations:** 1 Health Policy Research Group (HPRG), Dept of Pharmacology & Therapeutic, College of Medicine, University of Nigeria, Enugu Campus, Enugu, Nigeria; 2 Dept. of Health Administration & Management, Faculty of Health Sciences & Technology, College of Medicine, University of Nigeria, Enugu Campus, Enugu, Nigeria; 3 Department of Public Health, State Ministry of Health Dutse, Dutse, Jigawa State Nigeria; 4 Leprosy & Tuberculosis Relief Initiative (LTR), Jos, Plateau State Nigeria; 5 World Health Organization (WHO), TB Surveillance Office, Kano, Kano State Nigeria

**Keywords:** *Economic evaluation*, *Cost-Effectiveness analysis*, *Legacy Project*, *Leprosy case-detection*, *Cost per case*, *Nigeria*

## Abstract

**Background:**

The state of leprosy in Nigeria and the realities of post elimination era underscore the need for evidence- based cost-effective approach to early case detection for enhanced control and elimination of leprosy. This study evaluated the operational cost-effectiveness of a community delivered Legacy Innovative Project implemented to enhance leprosy case detection in northern Nigeria.

**Methods:**

Data were collected from an explorative cross-sectional study, undertaken in a practice setting among endemic communities in three states in northern Nigeria. Primary and secondary data were collected from the project, routine records and programme annual reports. Costs and effects were measured from both providers’ and patients’ perspectives, and outcome expressed as cost per new case detected. Incremental estimates of costs and effects of the project compared to routine practice were used to obtain the cost-effectiveness result, as incremental cost-effectiveness ratio (ICER). All costs were converted to the US Dollar at 2018exchange rate (N350 = US$1.00). Univariate sensitivity analysis was performed to evaluate uncertainties around the ICER.

**Results:**

The Project overall detected a total of 347 newly confirmed leprosy cases at a total annual cost of US$49,337.19, averaging US$142.18 per new case detected. Key cost drivers included routine meetings, which accounted for 28% of total expenditure, while Social Mobilization and Training/Workshop accounted for17% respectively. Findings were similar across the states. Overall, the Project dominated routine practice with ICER of US$(-17.73) per additional/new case detected, as a very cost-effective strategy. Sensitivity analysis reinforced the cost-effectiveness result.

**Conclusions:**

The Legacy Innovative Project demonstrated a more efficient and cost-saving approach to leprosy case detection. Findings present important information to policy and programmes for enhanced control and elimination of leprosy in related settings.

.

## Background

Leprosy remains a communicable disease that has been classified as neglected given the level of prevalence among the global population. On the basis of current global burden of the disease it was classified for elimination, defined as reducing the prevalence to less than one case per 10,000 populations [1]. Although Nigeria achieved the elimination goal of leprosy disease in 1998, the disease still persists as data suggest continuous spread with child cases remaining high at 9.0% while disability grade 2 (DG2) stays at about 13%, higher than 5% target [2, 3]. Meanwhile, based on the Leprosy Burden Score by the WHO [4], Nigeria has been recently categorized as a country of ‘high’ burden of leprosy [5], evidently due to pockets of relatively high endemic areas in the country. This raises concern about hidden cases and continuous spread of the disease as well as effectiveness of current methods in identifying early cases for effective control. This is despite the several measures implemented to control the disease with support from international agencies. The insidious nature of leprosy which takes between 2 and 5 years to manifest contributes to the difficulty in identifying the infection for prompt treatment, making early case detection critical to effective management and control [6, 7].

Hence, as a core activity in leprosy control and elimination, early detection of cases is crucial in achieving the objectives of control measures and prevention of disabilities. Delays in detection and subsequent treatment have serious implications for development of DG2, the permanent disability which is responsible for the social discrimination and stigmatization associated with leprosy disease [8]. Evidence has linked these delays to visible disabilities such that the longer the delays the more visible the disabilities [6]. Patients and health system factors have been identified to contribute to the delays in early case detection for subsequent treatment; such as culture and other socio-economic factors, etc. [9, 10].

New case detection of leprosy is also critical to ensuring the sustainability of the disease control, having been recognized as one of the most important components of secondary prevention in any disease control programme, in addition to case holding [11]. Consequently, greater impact of early detection of leprosy on the disease prevention is emphasized as a key control target to avoid progression to the late stage (DG2) that lead to permanent physical disability. In other words, any detection strategy capable of identifying more of early cases of leprosy will be more cost-effective and efficient in leprosy control. This will justify investment in such strategies to achieve the objectives of control measures. As a result, the timely detection of new cases of leprosy and prompt treatment with Multi Drug Therapy (MDT) among suspects in endemic countries becomes the key targets of strategies designed to reduce the burden of leprosy disease [12]. This makes early case detection and treatment of nerve damages the two main components of prevention of disability due to leprosy [10, 13].

The current Nigerian National Strategic Plan for Leprosy and Buruli is focused on improving current efforts at early case detection, to enhance the goal of reducing DG2 to 1 case per million population and eliminate child cases to zero [2], in line with global strategy updated in 2016 [5]. This called for the development of more effective and efficient approaches to identifying the disease early in its development process, for prompt treatment to prevent progression to physical disability and consequent social stigma. Previous efforts at this informed the review and evaluation of available detection methods to identify the most efficient option for improved results. This led to the evaluation of strategies, including passive case detection (PCD), active case finding through the mini Leprosy Elimination Campaign (Mini- LEC), and household contact examination (HCE)[7].These became necessary given the realities of post elimination era which requires evidence based cost-effective approach for more effective control of leprosy. Findings of the study led to the recommendation of a combination of strategies for improved results. Feasibility of this approach and the need for a more aggressive strategy led to the creation of the Leprosy Innovation Project (LIP), otherwise known as the Legacy Project, (LP). The project is a combination of strategies that incorporated active case finding approaches to case detection. This study was therefore conducted to evaluate the economic efficiency of the innovative Project implemented at the community level to enhance leprosy case detection in endemic areas of the three states, to inform policy for improved detection and elimination of leprosy in Nigeria. This would enable the achievement of the objective of reducing the DG2 to 1 case per million and eliminate disability among child cases to zero respectively, as contained in the current national strategic plan [2].

## Methods

### Study area and population

The study was conducted in Bauchi, Jigawa and Kano states, three neighboring/contiguous north-eastern and north-western states of Nigeria occupying a total land mass area of about 91,791Km^2^, with a 2018 combined population of over 25 million, based on the 2006 population projection. Mostly rural, the area has a predominantly young population (49.5%) below 15 years old, with limited access to basic sanitation [7]. Administratively the area has a total of 91 LGAs. In line with the Nigeria health care system, healthcare provision is centered on the Primary Health Care (PHC) system. Between the states, there are over 2, 750 primary, 75 secondary and 6 tertiary health care facilities.

Leprosy services are integrated into the National Tuberculosis and Leprosy Control Programme (NTBLCP) established in 1988. The two services are both supported by the Netherland Leprosy Relief (NLR) agency in Nigeria since 1988.The three states all achieved the WHO elimination target of less than one case per 10,000 population in 2000, even though challenges remained [7]. Although the WHO target of less than 1 case per 10,000 population was achieved as at the end of 2000 in the three states, the distribution of leprosy in the area shows wide geographical differences with some LGAs reporting high while others reporting low number of cases. This study took this variation into consideration by purposely selecting samples of 36 endemic communities where the project was implemented in the selected LGAs of the three states. This was based on annual reports of detection rates, for relatively high rate (endemic) areas. The project was implemented from Quarter 2 of 2017 to Quarter 1 of 2018 in the three states consecutively.

### Study design

A cross-sectional study was designed around leprosy contact levels, targeting endemic communities in the study area, to evaluate a one year operational cost-effectiveness of the innovative case detection strategy based on data available from 2015/2016. Using prevalence data from the NLTBCP 2015 records, 36 communities from 18 LGAs were classified as endemic, having more than 1 case per 10,000 population [2, 7]. The comparable endemic communities from the three contiguous states were then purposively selected based on implementation of the project/strategy. They were selected to also cover geographical locations where the project was implemented. Another criterion for selection included the presence of adequate number of MDT clinics for necessary referrals. Where there was no MDT clinic close to the endemic community, the nearest health facility was identified and the staff trained on Leprosy service provision.

Data for routine passive case detection were collected retrospectively for between 2015 and 2018. The legacy project data were collected from the implementation period of 2017 to 2018, for a 1-year comparative analysis of costs and consequences. Effectiveness data was measured in terms of the number of newly confirmed leprosy cases detected within the period. These comprised of all patients diagnosed and confirmed with either Pauci-bacillary (PB) or Multi-bacillary (MB) leprosy including children and other patients with disability grades 1 and 2. The MB is the more infectious strain of the bacteria responsible for over 90% of identified leprosy cases in Nigeria, and higher among child cases [2, 3, 14]. The study was designed to evaluate the Legacy Project as an intervention compared with the routine practice from neighbouring comparable communities as the control within the same LGAs.

## Description of interventions

### The legacy project set-up

Details of the project set-up and operation as implemented in the three states are described in the previous evaluation report [14]. The project started in April 2017.

Highlights of the innovative project include,

- Selection and use of volunteers/alternative healthcare providers or community leprosy workers (CLWs), 5 from each cluster, through community leaders and LGA TBLS supervised by the project team
- Advocacy visits to community leaders and ex-leprosy patients for sensitization of the project activities
- Sensitization and awareness campaigns to the community for the project activities
- Development and printing of coded referral cards and registers
- Training and orientation of volunteer health workers on roles and responsibilities
- Conduction of screening tests on suspects following examination of signs and symptoms
- Provision of incentives/as allowances to the volunteer workers for the project
- Provision of free drugs to suspects
- Supportive supervisions of the project activities were conducted at various levels
- Quarterly data reporting in line with the routine programme reporting. All registered cases are captured in the leprosy central register.
- Data validation was done at various intervals to ensure data quality.
- Quarterly review meetings were conducted at the end of every quarter with CLWs, GHCWs and the LGA TBLS in attendance

### Study sites and selection

These include.

- Selected high burden LGAs of intervention; 18 in all, from the three states, using 2 clusters of communities from each LGA (total of 18 LGAs, clusters of 36 communities).
- Five (5) Community health providers were selected from each cluster, making a total of 180 Community Leprosy Workers (CLW).
- Further activities included identification and training of GHWs in MDT clinics to serve as referral centers for Leprosy suspects where diagnosis and treatment of Leprosy could be made.

### Routine (passive) case detection (RCD)

The passive case detection method, which constitutes routine practice in this study, generally involves voluntary or self-reporting by patients with suspected leprosy, and the active case finding methods at which Leprosy suspects were identified by Community Leprosy Workers (CLWs) and referred to the MDT clinics using referral cards for diagnosis and treatment by trained health personnel. The routine practice method integrates leprosy services into primary health care as part of general healthcare services. It involves self-referral or referral by local health worker for suspected cases. Routine health education sessions are carried out by trained healthcare workers at the health centers. People with suspected signs are counseled and referred to leprosy unit or peripheral health centre. Suspicious cases are examined by specialized health workers at the health center. Leprosy diagnosis is confirmed by trained health workers on leprosy. As part of the main structure of healthcare provision, RCD has the capacity to integrate other healthcare components or services such as Tuberculosis (TB) and Immunization control programmes.. Highlights of the RCD activities include the engagement of general healthcare workers (GHWs) for the provision of leprosy services, regular staff training and supervision by state and local government Tuberculosis and Leprosy staff (LGTBLS and STBLS), visits to health facilities (voluntary reporting) by patients, social mobilisation and health education.

The PCD methods and the cluster approach to Leprosy case finding share two things in common. The use of MDT providers that diagnose Leprosy at the MDT clinics who are supervised by the Local Government TB and Leprosy Supervisors who visit the clinics to confirm the diagnosis and provide other technical and logistic supports to the GHWs providing the MDT services. In the intervention clusters/communities, the Community Leprosy Workers are linked to identified MDT clinics within the clusters. The GHWs in the MDT clinics have been given an orientation on the project including the filing of referrals from CLWs and use of the LP specific Recording and Reporting Tools.

### Conceptual framework

The cost-effectiveness study was based on identification and analysis of costs and effects of the two detection methods from the study sites, consistent with standard practice. All costs and effects were measured from both the provider’s as well as the patients’ perspectives. Effectiveness was measured in terms of number of new leprosy cases detected and outcome expressed as cost per case detected. Incremental approach, using the routine RCD as a reference was used to estimate the costs and effects of the project by comparing the innovative method (Legacy Project) against the routine practice; to determine the additional cost per new case detected by the project, as incremental cost-effectiveness ratio (ICER). Analysis was carried out for average cost-effectiveness (ACER) to enable decision-makers take alternative decision based on which method has a lower average cost-effective ratio. Analysis was carried out for the three states together as a contiguous area and then separately for each state as well as together for the three states as a contiguous area with similar leprosy burden.

### Data collection procedures

The evaluation utilized several methods of data collection in various ways: project records, interview (to assess patient travel cost, number of visits to healthcare facility to seek for care, waiting time, travel time) observation, and record review. Table 1 summarizes the data source and data collection methodology that was used to gather evidence for evaluation.

**Table 1:** Calculation methods and sources of data

*Cost category*	*Calculation method*	*Data source*
Personnel	Top down	Salary records (routine practice)
Training/workshop	Top down	Accounts records
Social mobilisation	Top down	Programme/Accounts records
Incentives	Bottom up	Programme records
Transport	Bottom up	Accounts records
Shared costs	Top down	Staff interviews/ programme records
Patients/family costs	Bottom-up	Questionnaire survey

### Cost data collection and analysis

Cost data were collected from expenditure records and reports. The costs of implementing services in the Legacy Project were identified and measured using the ingredient approach. Activity based data complemented the ingredient method. Bottom-up approach was adopted to estimate the economic costs, (where information on resource use and costs were available [15] which involved identification and valuation of all resources required in the detection of new leprosy cases from the relevant perspectives. Where detailed information about full resource use and unit costs was not available, top-down calculations was performed [7, 13]. The resources were first classified as capital and recurrent costs and measured accordingly. Capital costs were obtained by annualization of the capital items over their expected life-span. All costs were converted to the US Dollar (US$) at the 2017 exchange rate, (US$1 = N350), average exchange rates between 2017 and 2018, reflecting the period of the study [7].

In the project implementation, several areas of resource use or cost elements were identified and measured accordingly for the evaluation. These cost elements were classified as personnel, training/workshops, social mobilization, transport and routine meetings. Personnel include salaries, allowances and incentives paid to the workers. Various allowances such as transport, feeding, DSA, etc. attached to trainings/workshops or regular project meetings were assigned to these centers accordingly. Personnel costs were based on proportion of health worker/staff time devoted to the leprosy case detection and allowances paid in the process. Personnel cost is obtained by multiplying the total annual income of each health staff by the proportion of time spent by the staff in each method especially for routine practice. Salary data were collected from standard Nigerian payroll scale and were then allocated. Training and workshops comprised of short-term (recurrent) and long-term (capital) trainings and workshop costs ( basically for the routine practice (RCD). Costs of social mobilisation included such items as advocacy visits, IEC materials, radio/TV adverts and promotion. Capital items included vehicles, motorcycles, long-term training and start-up costs. They were annualised over their useful time periods and discounted at 3% rate based on World Bank recommendation, capturing their depreciated costs as opportunity costs of time.

The routine programme costs consisted mainly of personnel (salaries, allowances and staff benefits for all categories of staff), training and workshop (short and long-term), social mobilisation (public enlightenments, community outreach, patient education, IEC materials etc.), vehicles (costs of depreciation) and patient/family costs. The main cost elements for the Legacy/innovation project were allowances paid to field staff, (which was paid in the form of incentives as (they were not paid any form of salary), training and workshop, social mobilisation and incentives provided in the form of de-worming dermatological preparations given out to encourage attendance. The free drug preparations given the suspects were treated as incentives rather than to material and supply cost category. Other cost elements for the legacy project approach include advocacy visits and social mobilization, short-term training, allowances paid to field staff/supervisors and the incentives paid out to CLW for referred cases. However accuracy of these allocations was subject to availability of reliable data because resource use documentation was not detailed and properly defined (non-specific). Greater efforts were made in identifying and separating cost items for allocation to appropriate categories for analysis.

From patient and family perspective, cost included direct out-of-pocket expenses incurred in transportation and hospital/diagnostic fees, and indirect costs of travel time and hospital (waiting) time. Hospital fees were not charged for leprosy at the time for RCD (data not available). The cost of time (time loss) was based on the prevailing minimum subsistence wage rate in Nigeria during the period of analysis. Possible new rates given the expected increase in minimum wage was however used in sensitivity analysis to assess the impact on the study results.

Costs incurred by the patients at the household were collected from a structured interview targeting 50 outpatients from hospital/health facilities in the area of study, to determine the average costs of transportation, travel and waiting time for the patient seeking care at the health facilities or project site. The survey was also used to establish the average number of times each patient visited a healthcare facility in the course of diagnosing or seeking care for leprosy. A special questionnaire was designed and administered on patients attending outpatient clinics in the study areas to estimate patients’ transportation costs, hospital fees and travelling and waiting time spent on seeking care for the various options, especially in the routine method. Cost of patient and family time were based on minimum subsistent wage available for Nigeria at the time.

### Shared costs

Shared costs are important items to be appropriately measured in cost-effectiveness study so as not to under-estimate the costs of alternatives that benefited from shared resources. Some activities of the legacy project were shared in the course of the field activities, such as the provision of TB services at different magnitude. Costs incurred in the process were treated as shared costs. Consequently, cost data for some services were adjusted for leprosy programme at proportions that reflected the level of resource use for leprosy case detection. Hence, the cost of training was shared 60:40 between leprosy and tuberculosis case detections respectively based on the identified level of activities. This was similarly shared for allowances/incentives and other related activities. However, the costs of the shared items for the Legacy Project were only assessed in sensitivity analyses rather than in baseline. For the routine detection method, 30% and 40% personnel costs were estimated for state and local government supervisors respectively for case detection based on shared activities with TB and other leprosy services. Step-down approach was used to measure and allocate shared costs. Major proportions were however varied in a sensitivity analysis to explore the impact on the result.

### Start-up costs

Some cost items were generated at the beginning of some activities which are one-off and therefore expected to last for longer than 1 year over the life of the project. Such expenditures include trainings and the purchase of some materials. Although the project is being evaluated over the 1 year period of implementation, it is only appropriate that the economic component of the start-up items be used to truly reflect its opportunity cost, given that the value will roll over in the event of continuity. However, the need for annual engagement of new volunteers would require such trainings, since they are not permanent staff, hence not treated as such in the project.

All the data were tabulated and analyzed using the Excel spreadsheet (Version 2007). Costing worksheets were first created to collect relevant items for each method. The sheets contain the lists of likely resources used by each method. Data from the worksheets were then entered into the spreadsheet already programmed to calculate the required programme costs based on standard methods.

### Measures of outcome

The intervention project’s effect or outcome is the number of leprosy cases detected. As a discreet outcome, effectiveness of the study was measured as the number of new leprosy cases detected, to best capture the effect of the detection methods at the level of control. Study outcome was measured as cost per additional case detected. Although the use of DALY as an outcome measure in cost-effectiveness analysis is recommended by the WHO [16], it is more appropriate for preventive or treatment interventions where clinical outcome measure include health related quality of life [7]. Number of leprosy cases detected as the main output of this study is a discreet outcome measure which is best captured using cost-effectiveness measure.

### Analytical approach to CEA

Analysis of the costs and effects data is guided by the design of the study in which the methods were independently implemented in a mutually exclusive context. The Legacy Project was implemented as an independent intervention different from the routine practice rather than complementary service. The innovative strategy is then compared with routine practice to determine the alternative that generates more outcomes (leprosy case detection) at a given cost, demonstrating more value for money, exclusive of each other. This supposes that the differences in costs and effects data between the legacy project approach and routine practice are incremental to routine practice so that comparison of the costs and effects will be based on incremental cost-effectiveness ratio (ICER). ICER seeks to identify an alternative that replaces an existing practice in the form of mutually exclusive option. It measures the additional cost that would be required to achieve more superior benefits (health effects/case detection) than the baseline [17].

Thus

$$ICER= \frac{\mathrm{Total\,Cost }\,A -\mathrm{ Total\,Cost }\,UC}{\mathrm{Leprosy\,Cases }\,A -\mathrm{ Leprosy\,Cases }\,UC}= \frac{\Delta \mathrm{Cost}}{\Delta \mathrm{Leprosy\,Cases}}$$

where ‘A’ stands for alternative method (in this case the Legacy project) and UC stands for usual care (RCD, the routine practice).

The method that yields the lowest ICER value is considered the most cost-effective alternative. Hence, the study used the ICER criteria to identify the most cost-effective method to replace existing practice for leprosy case detection.

However, a measure of average cost-effectiveness ratio, (ACER) implies that the strategy that produces the highest number of outcomes at a given/constant cost generates the lowest ACER, making it the most cost-effective and preferred option [17]. This measure becomes relevant only in the absence of an existing practice; where comparison between alternatives will be based on the ACER.

The ACER values are however presented for complementary and comparative analysis.

### Sensitivity analyses

Many important parameters which are critical determinants of the study results showed certain level of variability/uncertainty with the potentials of affecting the study result. As a variability test for robustness, these variables were subjected to one-way sensitivity analysis to assess their impact on the results. They include the discount rate, accuracy of case detection rate for the routine method, allocation factors for shared costs, subsistent (minimum) wage. Routine meeting costs were included because they constituted major cost components. The cost-effectiveness values were recalculated using different values of these parameters in the sensitivity analysis.

## Results

### Leprosy cases detected/effectiveness estimates

Table 2 summarizes new leprosy cases detected by each state. From routine practice, annual total of 129 cases were detected between 2017 and 2018.This was made up of 10.4% child cases and about 5% DG2. The Legacy Project within the same 1 year generated a total of 347 new cases with about 9% child cases and 6% DG2.Child cases, a sensitive indicator of leprosy disease transmission [18] were similarly high for both methods at almost 10% each. MB cases accounted for over 82% of all the leprosy cases.

**Table 2:** Distribution of new leprosy case detection by state (April 2017 – March2018)

Strategy/State	Cases referred	Cases Examined	Cases Confirmed (New cases) 2017				Total cases		Total annual new cases
			PB	MB	DG2	Child cases	Qtrs 2–3 2017	Qtr1 2018	
Legacy project									
Bauchi (5 LGAs)	4,745	4,535	3	67	8	14	43	27	70
Jigawa (8 LGAs)	4,464	4,013	15	140	28	15	155	15	170
Kano (5 LGAs)	5,068	5068	0	97	8	0	97	10	107
*Legacy Project Total (18 LGAs)*	***14,277***	***13,616***	***18***	***304***	***44***	***29***	***322***	***51***	***347***
RCD states (in 18 LGAs)									
Bauchi (5 LGAs)	***-***	***-***	***-***	***-***	***-***	***-***	***-***	***-***	***33***
Jigawa (8 LGAs)	***-***	***-***	***-***	***-***	***-***	***-***	***-***	***-***	***41***
Kano (5 LGAs)	***-***	***-***	***-***	***-***	***-***	***-***	***-***	***-***	***55***
*RCD (in 18 LGAs)* *Total*	***-***	***-***	***-***	***-***	***-***	***-***	***-***	***-***	***129***

*RCD* Routine Case Detection, *LGA* Local Government Areas, *PB* Paucibacillary, *MB* Multibacillary, *DG2* Disability Grade 2, *Qtr* Quarter

### Programme costs

The summary of the annual costs of the legacy project is shown in Table 3 by category, presented for both the provider and patients/family perspectives. It shows the relative composition of the Legacy project’s resource inputs for leprosy case detection in the areas of implementation over the 1 year period. The information is presented for the total project and by individual states.

**Table 3:** Programme cost distribution for leprosy case detection 2017/2018 (Naira)

*Perspective*	*Cost category*	*Item*	*Routine (RCD)* *(18 LGAs)*	*Proportion/* *%age RCD*	*Bauchi* *(5 LGAs)*	*Jigawa (8 LGAs)*	*Kano* *(5 LGAs)*	*Legacy Project* *(18 LGAs)*	*Proportion/ % Legacy Project*
*Provider*	*Recurrent*	Personnel/allowances	12,661,488	0.69	730,000	848,750	135,000	1,713,750	0.10
		Training/workshop	513,083	0.03	709,500	1,525,400	669,000	2,903,900	0.17
		Social mobilization	3,153,427	0.17	1,118,500	1,054,802	758,745	2,932,047	0.17
		Transport	361,602	0.02	245,000	260,000	135,000	640,000	0.04
		Material supplies	115,104	0.01	708,345	316,000	351,600	1,375,945	0.08
		Meetings	1,172,762	0.06	1,086,000	1,578,000	2,102,200	4,766,200	0.28
		Incentives	0	0.00	560,000	1,325,028	846,800	2,731,828	0.16
	Capital Cost	Start-up costs	0	0.00	0	0	0	0	0
		Training; long-term	21,743	0.00	0	0	0	0	0
		Vehicles	246,578	0.01	0	0	0	0	0
		*Subtotal*	*18,245,788*	*0.99*	*5,157,345*	*6,907,980*	*4,998,345*	*17,063,670*	*0.01*
*Patient & Family*	Direct	Transportation	107,503	0.01	0	0	0	0	0
		Hospital fees	0	0.00	0	0	0	0	0
	Indirect	Hospital time	7,087	0.00	6,265	8,732	5,484	20,482	0.001
		Travel time	107,503	0.01	40,100	88,306	55,458	183,865	0.01
		*Subtotal*	*222,093*	*0.01*	*46,366*	*97,038*	*60,942*	*204,346*	*0.01*
		*Total (N)*	*18,467,882*	1.00	*5,203,711*	*7,005,018*	*5,059,287*	*17,268,016*	*1.0*
		*(US$)*	*52,765*	*1.00*	*14,868*	*20,014*	*14,455*	*49,337.19*	*–*

*US$1* = *N350*

Overall, the Legacy Project generated a total cost of US$49,337.19 at a unit cost of US$142.18 per new leprosy case detected. The routine project meeting accounted for the highest proportion of total expenditure at 28%. It was followed by both social mobilization and training/workshop components which contributed 17% respectively. Incentives, as a major part of the project given to both the community leprosy volunteer workers and patients contributed 16% of the total implementation cost. At the state level, the project in Jigawa state implemented in 8 LGAs, generated the highest total cost of US$20,014compared to Bauchi and Kano states where the project was implemented in only 5 LGAs respectively, with similar annual cost/expenditure of about N5.00 million each. In each state the routine meeting expenses accounted for the highest proportion of total expenditure at over 20%, but most in Kano where up to 42% of the expenditure was spent on the routine programme meetings. Personnel was not a major cost driver in the project implemented at the community level where focus was on incentives paid to volunteers, compared to routine practice where personnel cost accounted for up to 70% of the total cost. In the Legacy Project, personnel cost was limited to the state and local government supervisors who were paid a proportion of allowances attributed to the project activities. The high cost of social mobilisation was also due to the number of activities involved in mobilizing communities for leprosy detection such as advocacies, education, communications etc.

As a community delivered project, based on active case finding, resources used in the Legacy Project are generated mostly as programme costs, unlike the facility based routine/passive case detection method where resources are generated mostly as capital and recurrent expenditures with significant overheads. Very little capital costs were generated by the Legacy Project in terms of initial training workshop conducted mostly for recruited CLWs who are engaged as volunteers and orientation trainings to the GHWs/LGATBLS.

Patient/family costs contributed only 1% of the total cost of the project, calculated from patients’ travel and waiting times as the patients still had to visit and wait to be served. Average transportation costs to and from health care facilities was estimated at $1.32 per patient while on the average a patient visits healthcare facilities 1.5 times before being diagnosed for leprosy, ranging from 1 to 4 times. Average travel and waiting times totaled 92 min (61 min and 31 min respectively), which translates to approximately US$0.41 per patient based on the minimum subsistent wage rate in Nigeria at the time.

### Cost per case detected

This indicator points to the relative efficiency of the project overall and in the respective states based on the cost expended in detecting one leprosy case. Table 4 shows that overall, the Legacy Project which yielded a total of 347 new leprosy cases within the year under study at US$49,337.14, averaging US$142.18 per new case detected, compared to routine practice at US$409.03 per case. The project in Bauchi state was implemented at the highest cost of US$212.40 per new case detected during the period, higher than the overall average, while Jigawa posted the lowest cost per case at US$117.73, suggesting the most efficient use of resources among the states. It was followed by Kano state at US$135.09per new case detected. Overall, cost per case of the Legacy Project was better compared to routine practice which detected lower number of new leprosy cases at the respective areas.

**Table 4:** Distribution of cost per leprosy case detected between the states

*Detection method*	*Leprosy cases detected*	*Annual total costs* *(N)*	*Cost per case detected*	
			***(Naira)***	***(US$)***
*Legacy project*				
Bauchi state (5 LGAs)	70	5,203,710.69	4,338.72	212.40
Jigawa state (8 LGAs)	170	7,005,018.45	41,205.99	**117.73**
Kano state (5 LGAs)	107	5,059,287.02	47,383.06	**135.09**
*Total Project (18 LGAs)*	*347*	*17,268,016.16*	*49,763.74*	*142.18*
*Routine case detection* *(RCD)*				
Bauchi state (5 LGAs)	33	5,431,730.26	155,453.55	**444.15**
Jigawa state (8 LGAs)	41	7,604,421.64	175,169.61	**500.48**
Kano state (5LGAs)	55	5,431,729.74	93,272.13	**266.49**
* Routine Case Detection* *(18 LGAs) Total*	*129*	*18,467,881.64*	*143,161.87*	***409.03***

*LGA* Local Government Area, *RCD* Routine Case Detection

#### Cost-Effectiveness estimates; base case results

Table 5 presents the estimated incremental cost per case detected by the Legacy strategy compared with the routine practice over the 1 year period. In the base case, the overall Legacy strategy produced an ICER of US$(-15.73) per additional case detected, which represents a gain in cost per additional leprosy case detected compared to routine method. In other words, the project makes a gain or savings of US$(-15.73) for any additional leprosy case detected beyond the routine practice. At the state level, findings show that the project was most cost-effective in Kano and Jigawa states where the ICER results were also negative, US$(-3.88) and US$(-3.92)respectively. However, in Bauchi state the ICER was US$5.69 making it the least but very cost-effective finding, in which an extra cost of US$5.69 is paid for one additional leprosy case detected (Table 5).

**Table 5:** Cost-effectiveness Results/Estimates

Detection method	Total Cost	Cases	ACER		*ICER*	
			*Naira*	(US$)	Naira	*(US$)*
Legacy overall	17,268,016	347	*49,763.74*	142.18	−5,503.97	***−15.73***
Bauchi state	5,203,711	70	74,338.27	212.40	1,993.07	***5.69***
Jigawa state	7,005,018	170	41,205.99	117.73	−1,371.59	***−3.92***
Kano state	5,059,287	107	47,283.06	135.09	−1,359.23	***−3.88***

*ICER* incremental cost-effectiveness ratio, *ACER* Average cost-effectiveness ratio

### Sensitivity analysis

The results on the CER of varying the values of major parameters which showed significant uncertainties are presented in Table 6. Shared cost proportions, subsistence wage, discount rate and case detection rates for the routine method (due to uncertainty in data documentation) did not significantly alter study results but reinforced the very high cost-effectiveness findings. When the shared costs proportions was made 60:40% between leprosy detection and tuberculosis, the project total and average costs reduced by over 31%, vastly increasing the cost-effectiveness of the project. Reducing the cost of the routine practice method due to uncertainty of data documentation increased cost-effectiveness of the results. The results were similar in all the states. Variation of case detection rate, routine practice data, and project’s routine meeting costs did not significantly change the results. Findings suggest that from both providers and patient/family perspectives and at all contact levels demonstrated a very cost-effective approach for identifying new leprosy cases for effective prevention and control of leprosy in Nigeria.

**Table 6:** Results of Sensitivity Analysis of selected parameters (in US$)

*Parameters*	*Scenarios*	*Change in results*	*Legacy* *overall*	*Bauchi* *state*	*Jigawa* *state*	*Kano* *state*
Shared costs	60:40%	Program Cost (%)	−31.5%	−32.5%	−30.8%	−31.2%
		*ICER*	US$(−86.7)	US$(−125.2)	US$(−51.68)	US$(−89.60)
RCD cost	−25%	*ICER*	US$44.8	US$104.73	US$35.85	US$66.58
Case detection rate	50%	Program Cost (%)	0	0	0	0
		*ICER (%)*	$-23(43)	$10(84)	$-4.5(19)	$-8.4(112)
Minimum wage/salary						
	18,900	Program Cost (%)	19	16	18	17
		*ICER (%)*	0	0	0	0
Meeting Costs	−25%	Programme costs				
		*ICER*				

*RCD* Routine Case Detection, *ICER* Incremental Cost-Effectiveness Ratio

## Discussion

The study evaluated the economic efficiency of innovative Legacy Project implemented to improve the detection of leprosy cases in endemic areas of three northern states in Nigeria. With the impact of the project in terms of the number of identified new leprosy cases determined, this study evaluated the economic implications of the project by measuring the resource utilization in relation to the project outcomes, to determine the economic efficiency. This has become necessary to determine the feasibility of the project and decision to scale-up and adopt beyond the area of implementation, among other accountability goals.

Results of the cost analyses show that the implementation of the Legacy project yielded a total economic cost of US$49,337.19 to detect up to 347 newly confirmed cases of leprosy in the study area within the 1 year period. This translates to an average cost of US$142.18 per newly confirmed case of leprosy, compared to the Routine practice which averaged $409.03 per new case detected. Apparently while the total costs of the two strategies are not significantly different from each other, the Legacy project is able to identify up to three times the number of cases detected through the Routine practice. In other words, at any given cost the Legacy project detects about three times the number of leprosy cases detected through Routine practice, demonstrating greater efficiency of resource utilization. In the project resource utilization is mostly strategic, linked to leprosy case finding. As an active case finding strategy which is field-based and community oriented activities are directly focused on leprosy case finding, leading to resources allocated to the relevant activities according to the level of output. This is not the case in routine practice where personnel accounted for the commanding share (70%) of total cost (consistent with previous findings in Nigeria where personnel account for about 80% of total cost of service delivery [19]) due to salary payment method which is input-based payment system not linked to performance / leprosy case detection, hence inefficient [20]. In contrast personnel accounted for only 10% of total cost under the Legacy Project as resources were allocated towards incentives and bonuses paid to CLWs according to performances, in the form of referral of leprosy suspects. All activities and therefore resource allocations under the LP are linked to performances related more case findings leading to cost savings most apparent in personnel costs reduced to 10%. This is a reflection of efficiency in better use of resources and more case findings.

This cost finding (US$142.18) is far lower than a recent study that reported a mean cost of US$424 per new leprosy case in Nepal [21]. Similarly, a previous study in India by Ganapati et al. [22] reported a mean cost of US$120, which is very similar to this study. The differences can be attributed to many factors ranging from geographical settings, detection rates, prevalence rates, foreign exchange rate (which does not often reflect the true economic parity between countries compared to Purchasing Power Parity, not available at the time) and importantly the efficiency of resource allocation and utilization, among others. These would also explain the differences in the mean cost per case detected between the three states where the project was implemented, using the cost findings as an indicator of relative efficiency. Consequently, the project in Jigawa state which returned the lowest cost per leprosy case detected at US$117.73 is probably more efficient compared to the other two, where Bauchi state posted the highest cost per case at US$212.40. Nevertheless, these rates which informed the overall LP average value, fall within the global context in leprosy case detection. However, apart from resource allocation and utilization, lower prevalence or reduced chances of increased leprosy case detection may have informed the differences between the states. For instance, some reported challenges that affected the implementation of the project, led to temporary suspension in some of the states during the third quarter, thereby reduced chances of case detection. In particular, lack of incentives to both volunteers and patients affected output due to temporary suspension of the project when the incentives were exhausted. This underscores the importance of sustainability and adequate supply of incentives for enhanced efficiency of the process. In addition, better and more effective use or allocation of resources between the states would have improved the relative efficiency.

The high cost of implementation in Bauchi state can easily be attributed to the very low case detection rate which was lowest at 70 compared to the other two states. There are two possible explanations to this finding. This may be largely due to low prevalence of leprosy in the state, considering that the state spent a similar amount of N5 million in 5 LGAs, just like in Kano state. Otherwise, inefficiency of resource management or allocation may similarly explain the high cost per case, or even a combination of the two. Apart from low case detection rate, there was also a high cost allocation to personnel (14%) which was not result-driven compared to incentives that received only 11%. However, in Jigawa and Kano states which posted lower cost rates, incentives accounted for 19% and 17% respectively, while personnel received 12% and 3% respectively. Hence, it was no surprising that the two states posted more cases than Bauchi, given that incentive attracted more patients in those states. Reallocating more resources to incentives therefore would boost case detection, and in consequence reduce the cost per case detected.

The cost-effectiveness results (ICERs) of this study, at overall cost of US$(−15.73) per additional leprosy case detected have demonstrated that the innovative Legacy Project is not only very cost effective strategy that represents good value for money, it is cost saving. This implies that for every new leprosy case detected, there will be a saving of about US$15.73 [7, 15, 16].

Apart from the previous study on CEA of three leprosy case detection methods in the area [7], preceding this study, no study has been documented on the cost-effectiveness of case detection methods. Hence, it was not possible to make comparisons. However, published studies on cost-effectiveness analysis of leprosy interventions are very limited [7, 23], and none is related to case detection, making comparison of findings difficult. A study in Bangladesh [24] evaluated the cost-effectiveness of chemo-prophylactic intervention with a single dose Rifampicin in household contacts of new leprosy patients, which resulted in a CER of US$158 per additional case prevented.

As described above analyses of resource use and allocation in this study largely explained the cost-effectiveness results. Findings suggest that resources were more optimally and effectively used to achieve the desired objective of detecting leprosy cases compared to routine practice, where costs per case are very high, with overall average of US$409.03. In the routine practice as stated earlier, personnel accounted for almost 70% of cost of detection at US$280.0, while in the Legacy Project it accounted for only 10%. This may make personnel appear largely redundant in the routine health system while the use of community based volunteers for the project, who were paid through allowances and bonuses as incentives, was very optimal leading to substantial cost savings. They were not only very active in the service; they also contributed to enhanced mobilization of leprosy suspects. The impact of social mobilization through campaigns and community advocacy for enhanced awareness and subsequent increased case findings explains why it commanded a leading proportion of the total cost of the innovative project. The high proportion of routine meeting cost as the leading cost driver can be attributed to the number of activities that were undertaken during the period which are resource-driven, such as refresher trainings, data collection and review activities, identification of operational challenges among others.

The results of the sensitivity analyses demonstrated the robustness of the cost-effectiveness findings also evidenced at the state levels and from both provider’s as well as patients’ perspectives. The perspectives of the study were necessary to analyse the costs and benefits data from broader viewpoints that include the patients/family for more balanced and comprehensive decisions, such as considering the need for subsidy. The sensitivity analysis also demonstrated substantial increase in the efficiency of the project when costs are shared, through reduced costs and increased cost-effectiveness ratios. This suggests that implementing the innovative project with related community based programmes such as TB control will enhance cost savings and efficiency. Similarly, reallocating resources from some activities such as routine meetings to input items like incentives for volunteer workers and patients’ free drugs will drive more outputs. Other parameters showed no significant impact.

The CER falls within the WHO’s category of studies classified as highly cost-effective interventions; being less than three times the Gross Domestic Product (GDP) [7].The very low unit cost per case detected at US$142 indicates that resource use is lower or more efficient in detection of more cases, suggesting that it will be easier to scale-up to achieve increased case finding rates, to enhance eradication of leprosy. The study will justify the adoption of this innovative strategy as a routine method in many countries in line with WHO recommendation [25]. Many factors can explain the findings, such as implementation costs per case which is one of the lowest per unit of output (per case detected). Compared to the existing health facility based approach, the method requires less manpower. Results of the sensitivity analyses reinforce the very high cost-effectiveness of the innovative method in the study area. However, although the study has demonstrated the cost-effectiveness of the Legacy Innovative Project which may have also been underestimated given certain considerations and challenges in implementation, the strategy could be incorporated into routine practice as part of the routine health system to leverage on available resources where those resources, in particular personnel are underutilized. This implies that smarter use of resources to reduce cost without compromising quality will increase the cost-effectiveness of the innovative strategy for enhanced efficiency in leprosy control.

As a follow-up to the previous study on the cost-effectiveness analysis of three leprosy case detection methods in the area [7], this study becomes the second of two published studies to date contributing to literature on the cost-effectiveness of leprosy case detection.

### Limitations of the study

The study experienced some limitations that need to be highlighted for better use of information. However, efforts were made applying standard approaches to improve the reliability of data. Cost studies such as this is always fraught with challenges, in particular due to paucity of data in public health programmes. Poor and inadequate documentations led to projections that may affect the true value of resource use. However, efforts were made to enhance reliability of the study findings based on standard approaches [15], such as subjecting the results to extensive sensitivity analyses with uncertain variables. The retrospective data from routine practice is subject to recall biases and resource use data were not very detailed and specific, resulting in greater efforts at separating and allocating resources. The gap in data availability may have resulted in some costs not adequately captured. Hence, accuracy of cost allocations may require the need for field study for more accurate results.

Effectiveness data analysis did not consider future impact beyond a 1-year period as the mix of leprosy cases detected have varying benefits from prevention of progression to permanent disability. The higher the number of early disease cases the greater the potential benefits of being prevented from progression to permanent disability, with MDT treatment. The use of DALY as a measure of outcome will involve the measure of utility for each leprosy grade detected such that the higher the number of early cases the higher the utility and hence the benefits. Lack of data on utility values and inadequate information on the proportion or distribution of leprosy categories limited this approach. However, this may not have changed the study findings since the methods yielded similar proportions of child and DG2 cases.

Lastly given the dynamic nature of leprosy transmission as a communicable disease, the study did not capture the benefits of secondary prevention beyond 1 year, but only based on the analysis of primary prevention. Hence, a dynamic model would have captured the benefits of further prevention beyond the primary cases but this was not possible as information on the long term impact of prevention is not available. This would have increased the cost-effectiveness of the legacy strategy.

Economic evaluation of health care programmes or interventions such as this provides the bases for informed critical decisions or policy with insights into appropriate allocation of resources. Hence, planning and conducting these studies require careful consideration of methodological differences because of the implications on the validity of study findings and their generalizability. The focus of economic evaluation on the cost of an intervention to produce a desired health outcome relies significantly on modeled evidence- based assumptions due to data limitations. Hence, the increasing number of interventions with known health impact calls for more evidence on ways of operationalizing delivery of these health programmes to ensure high quality and cost-effectiveness.

## Recommendations

1. *Adoption or integration into routine practice*. Having demonstrated a very cost-effective and cost-saving strategy, the Legacy Project is highly recommended for adoption to complement routine practice to boost leprosy case finding for enhanced control and elimination of the disease.
2. *Combination with other related healthcare services:* Resource utilisation will be highly enhanced as indicated in Sensitivity Analysis if the project is combined with related community based health services such as TB and immunization programmes. Sharing the project resources with related activities will boost efficiency for increased outcome
3. *Develop a protocol for more effective data documentation:* Poor and inconsistent documentation encountered in the study common in our setting, underscore need to develop appropriate template and tools for data documentation to ensure accuracy of information for effective monitoring and evaluation, and planning purposes.
4. Given its efficiency in greater leprosy case detection at lower costs, the study strongly recommends the adoption of the innovative strategy by relevant agencies involved in leprosy programme to sustain the control and elimination.
5. *Implement a field study to improve accuracy and reliability of study findings*. The many limitations of data collection in the study, revealing gaps in data availability and documentation calls for a field study to correct these gaps and enhance the accuracy of data and reliability of study findings.

## Conclusions

From both providers’ and patient’s perspective, evidence suggest that the community delivered Legacy Innovative Project is a very cost-effective and low cost strategy for leprosy case detection. This makes the project a very attractive strategy to be adopted by policy makers for improved leprosy case detection for enhanced elimination. Community involvement through their leaders and volunteers was a major contribution to the success, reducing cost while increasing mobilization for identifying suspects. The effectiveness can be further enhanced by implementing recommended strategies such as community based health related activities including in particular TB, while addressing many identified lapses in the implementation process. These efforts will further reduce costs while enhancing case detection rates. The cost of implementation offers the best option for scale-up to increase coverage and achieve optimum control of leprosy in Nigeria and similar settings.

## Data Availability

The datasets generated or analyzed during the current study are available from the corresponding author on reasonable request.

## Abbreviations

ACER: Average cost-effectiveness ratio
CEA: Cost-effectiveness analysis
CER: Cost-effectiveness ration
CLW: Community leprosy worker
DSA: Daily subsistence allowance
DG2: Grade 2 disability
GHW: General healthcare worker
HCE: Household contact examination
ICER: Incremental cost-effectiveness ratio
IEC: Information and education communication
LEP: Leprosy elimination programme
LGA: Local government area
LIP: Legacy innovation project
LTR: Leprosy NS tuberculosis relief
MDT: Multi-drug resistance
NLR: Netherlands leprosy relief
NLTBCP: Nigerian leprosy, tuberculosis control programme
PCD: Passive case detection
PHC: Primary healthcare
TBLS: Tuberculosis and Leprosy Service
WHO: World health organization
